# Influence of the Composition of Provisional Luting Materials on the Bond Strength of Temporary Single-Tooth Crowns on Titanium Abutments

**DOI:** 10.3390/ma17174239

**Published:** 2024-08-27

**Authors:** Christina Maubach, Heike Rudolph, Arndt Happe, Ralph G. Luthardt, Katharina Kuhn, Sarah M. Blender

**Affiliations:** 1 Center of Dentistry, Department of Prosthetic Dentistry, Ulm University Hospital, 89081 Ulm, Germany; christina.maubach@uniklinik-ulm.de (C.M.); heike.rudolph@uniklinik-ulm.de (H.R.);; 2 Private Practice, 48143 Münster, Germany

**Keywords:** provisional dental materials, temporary dental cements, luting agents, temporary restorations, bond strength, titanium abutment, retention, artificial aging, short- and long-term water storage

## Abstract

In addition to zinc oxide-based cements, resin-based materials are also available for temporary cementation. The aim of this in vitro study was to determine the influence of the different material compositions on temporary bonds. In nine test series (n = 30), temporary bis-acrylate single-tooth crowns were bonded onto prefabricated titanium abutments with nine different temporary luting materials. After simulating an initial (24 h, distilled water, 37 °C), a short-term (7 days, distilled water, 37 °C) and a long-term provisional restoration period (12h, distilled water, 37 °C; thermocycling: 5000 cycles) in subgroups (n = 10), the bond strength was examined using a combined tensile–shear test. Statistical analysis was performed by univariate analysis of variance or a non-parametric Kruskal–Wallis test, followed by post hoc tests. Of the three resin-based materials, two showed significantly higher bond strength values compared to all other materials (*p* < 0.001), regardless of the storage procedure. The resin-based materials were followed by eugenol-free and eugenol-containing zinc oxide materials. Significant intragroup differences were observed between the composite-based materials after all storage periods. This was only observed for some of the zinc oxide-based materials. The results show that under in vitro conditions, not only the composition of the temporary luting materials but also the different storage conditions have a significant influence on temporary bonds.

## 1. Introduction

Dental implants can be restored with fixed temporary restorations during osseointegration (immediate loading) or after implant exposure following a submerged healing period to restore aesthetic and functional properties. The surrounding soft tissue can be contoured according to the requirements of the permanent restoration by shaping the temporary restoration correspondingly and thus can positively influence the treatment outcome, especially in aesthetically challenging regions. At the same time, the patient’s expectations regarding the shape, color and design of the subsequent permanent restoration can be explored and, if necessary, be varied and adapted with little effort. This ensures patient comfort and simplifies the communication with the dental laboratory [1,2,3,4].

Temporary luting materials are used to retain the restorations in situ for a certain period of time. The duration of the provisional restoration period can range from classic short-term (from a few days up to three weeks) to a more demanding long-term period (several months) depending on the clinical situation. Ideally, the temporary luting materials retain the restorations in the correct position for the entire provisional restoration period. This prevents mechanical irritation of the surrounding gingiva and soft tissues due to any necessary corrections or recementation. However, if the treatment procedure requires the temporary restorations removal for certain steps it must be easy to remove [5].

The most popular representatives of temporary luting materials for provisional implant-supported restorations are zinc oxide–eugenol cements, which are mixed from two pastes [6]. The advantages are the low costs, their simple handling properties, the easy removal of remains and the long clinical experience with the use of eugenol-containing materials. While the eugenol component of these cements has advantageous pulp anaesthetic effects and antibacterial properties for teeth [7,8], these characteristics are irrelevant for implant-supported restorations.

However, residual eugenol components in dentin tubules or on abutment or restoration surfaces inhibit the chemical setting reaction of adhesive and self-adhesive luting materials for permanent restorations [9,10,11]. To avoid this problem, eugenol-free zinc oxide cements can be used in which the eugenol component has been replaced by an organic acid [12,13]. In contrast to eugenol-containing materials, there is less clinical evidence for this composition, but it has become popular for the temporary cementation of implant-supported restorations [6,12]. Zinc oxide cements achieve their bonding by mechanical bonding forces, such as friction and wedging, which is why a sufficient retention form of preparation or abutment is required for adequate retention. Due to their water-soluble properties, they are less resistant to compressive and tensile forces and therefore need to be renewed after a certain period of time [14,15].

Like definitive luting materials, there are temporary materials that also have adhesive properties in addition to their macro- and micro-mechanical bonding components. Their chemical bonding component allows a direct interaction of the luting material with other surfaces, resulting in better retentive properties [14,15]. These materials are similar in composition to filling materials and are based on resins or methacrylates, which makes them more resistant to water solubility and thus positively influences their retention properties [16]. Another advantage is their translucent and tooth-colored appearance. In contrast to zinc oxide cements, with their very opaque and whitish color, methacrylate-based materials provide aesthetically pleasing results and at the same time sufficient retention, even in temporary restorations of low-layer thickness [17,18].

While standardized requirements for the manufacturing of the test specimens, setting time, compressive strength, film thickness and arsenic release of zinc oxide-based temporary luting materials exist [19], there is no guideline for evaluating the bond strength of temporary luting materials, regardless of their composition. Nor are there any reference values for determining adequate temporary or permanent bonding of luting materials. Due to the limited evidence on the use of methacrylate-based temporary luting materials, a comparison with classic zinc oxide cements in terms of retention can thus hardly be accomplished [12].

Thus, the aim of this study was to investigate the bond strength of different temporary luting materials when bonding temporary bis-acrylate crowns on titanium abutments. This study aimed to evaluate the influence of different material compositions (zinc oxide–eugenol cements, zinc oxide eugenol-free cements, resin-based materials) on the temporary bond strength as well as the bonding performance of the individual materials during the simulation of different temporary storage periods (initial, short-term, long-term).

## 2. Materials and Methods

The bond strength of temporary bis-acrylate single-tooth crowns to prefabricated titanium implant abutments was analyzed in nine test series (n = 30 each), one series for each of the nine differently composed temporary luting materials (Table 1). Three products were selected from each of the three temporary luting material classes, (1) zinc oxide-based with eugenol, (2) eugenol-free zinc oxide-based and (3) resin-based and stored according to the manufacturers’ instructions. When choosing the materials, care was taken to consider all material classes offered by a manufacturer and to select materials that have already been described in the literature to ensure comparability. Appendix A Table A1 lists the composition of the materials as specified by the manufacturer.

**Figure 1:**
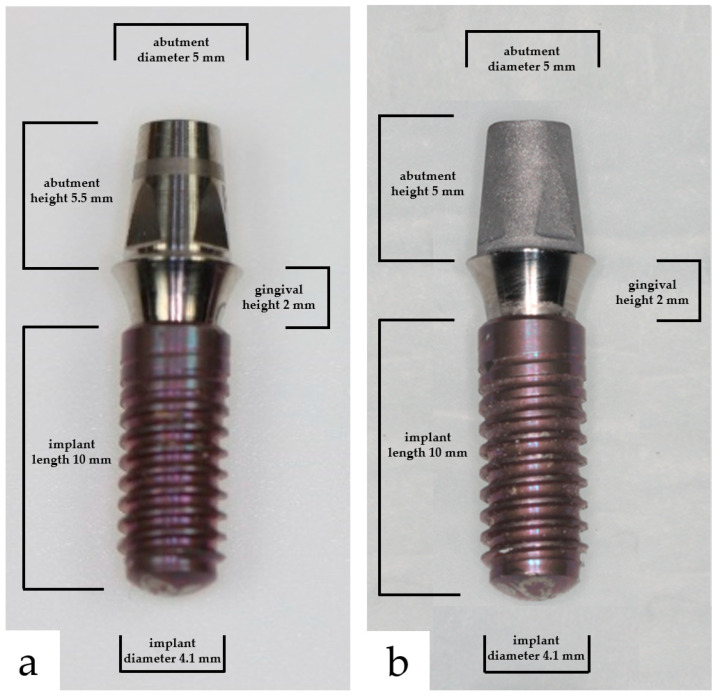
(**a**) Prefabricated abutment screwed onto the training implant before surface pretreatment; (**b**) the abutment after shortening to a height of 5 mm and after sandblasting of the bonding surface with aluminum oxide.

The prefabricated titanium abutments (RC Cementable Abutments, diameter of 5 mm, gingival height of 2 mm, abutment height of 5.5 mm, titanium, Straumann GmbH, CH) were shortened to 5 mm. Although this was irrelevant for the study presented here, it will enable us to better compare the results with our own existing or future data with other material combinations or abutment materials that are different but of the same diameter and height. The bonding surface of the abutment was pretreated for better retention. For this purpose, the entire bonding surface was painted with a waterproof color marker and then sandblasted with aluminum oxide with a grain size of 50 µm at 2.5 bars at a distance of 10 mm until the color marker was completely removed. Afterwards, surfaces were cleaned with 98% ethanol in an ultrasonic cleaner and then dried with oil-free air. The pretreated abutments were then screwed onto training implants with a diameter of 4.1 mm (training implant RC Bone Level, implant length 10 mm, Straumann GmbH, CH) (Figure 1a,b). The four parallel surfaces designed by the manufacturer on the lower half of the abutment are intended to provide sufficient retention. The abutments’ screw channel was occlusally sealed with a foam pellet, bonding adhesive (Optibond FL, Kerr Corporation, Orange, CA, USA) and composite (Venus Diamond, KULZER GmbH, Hanau, Germany).

**Figure 2:**
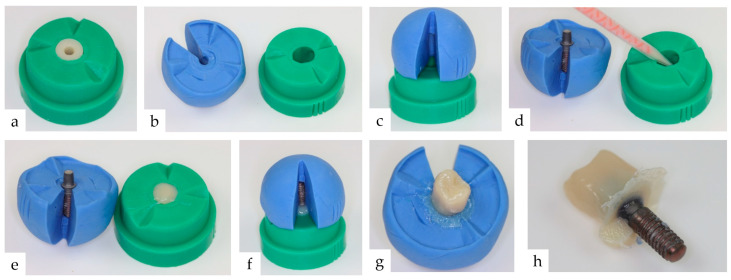
Simulated chair-side procedure for the fabrication of the provisional crowns with identical geometry and dimensions: (**a**) A master crown was fabricated according to the necessary criteria for the test setup and duplicated with a two-part silicone mold (green: crown outer surface) (Dublisil 15, Dreve Dentamid, GER). (**b**) The second part of the silicone mold (blue) was used to transfer the crowns’ position on the implant with the screwed-on abutment (Silagum, DMG GmbH, Hamburg, Germany). (**c**) Three small parallel lines were grooved into the outer surface of both mold parts in a corresponding position to check the exact positioning of the assembled mold parts. (**d**) To produce the single-tooth crowns, the temporary restoration material was filled into the first silicone mold (green). (**e**) The implant–abutment complex was placed into the second silicone mold (blue). (**f**) The blue mold was reassembled with the first resin-filled mold (green) and the correct position was verified using an inspection slot. (**g**) After the setting of the resin, the green silicone mold was removed (**h**) and the crown was finalized.

The bis-acrylic resin single-tooth crowns (ProTemp™ 4, 3M ESPE, Neuss, Germany were fabricated individually for each abutment prior to bonding using a simulated chair-side procedure. To produce single-tooth crowns in the shape of an upper premolar and with the same geometry and dimensions for all test specimens, a silicone mold (Dublisil 15, Dreve Dentamid GmbH, Unna, Germany of a prefabricated master crown was made (Figure 2). The duplication technique ensured the production of identical single-tooth crowns for each abutment.

**Figure 3:**
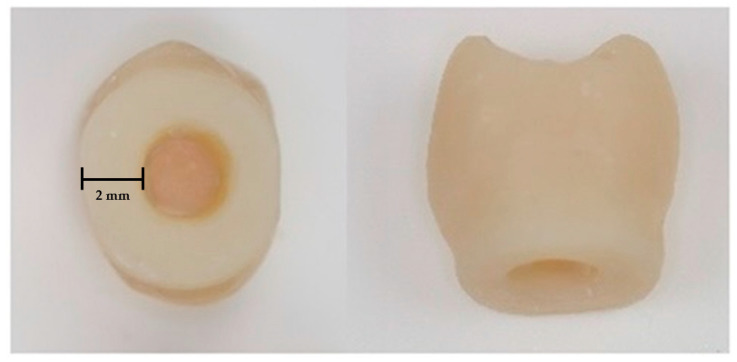
Chair-side single crown fabricated in the shape of an upper premolar with an overcontoured marginal area of 1.5–2 mm.

**Figure 4:**
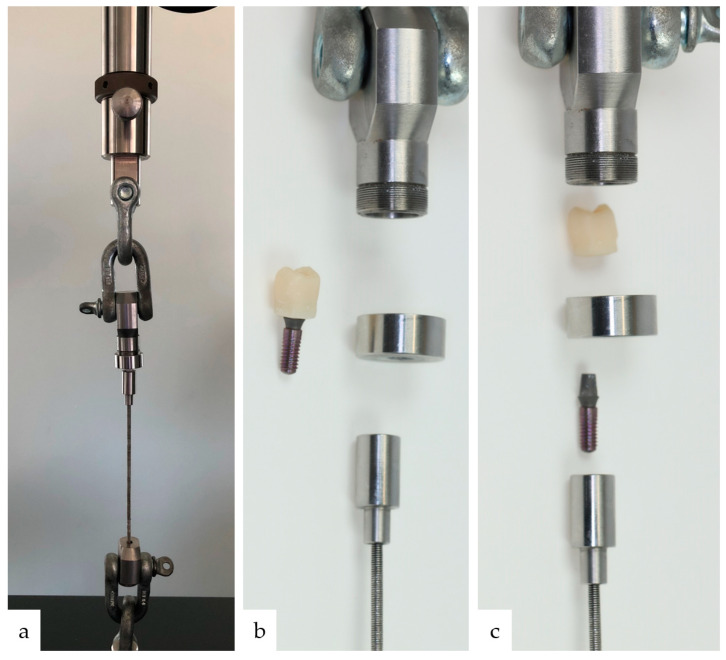
(**a**) shows the mounted test specimen in the universal testing machine. Since the internally positioned crown-implant specimen cannot be seen, the test setup is shown in more detail in Figures (**b**,**c**): **Top**: upper shekel with upper part of metal holding box; **Middle**: crown-implant specimen and lower part of the holding box with center hole at the bottom; **Below**: the internal thread to be screwed onto the implant part of the specimen and the threaded rod attached to the lower shekel. The overcontouring of the crowns’ marginal area prevents it from sliding through the bottom hole of the holding box.

In contrast to the clinical procedure, the crowns were overcontoured by 1.5–2 mm in the marginal area (Figure 3) to keep the specimens in place within the test setup’s mounting box (Figure 4).

Prior to bonding, the surface of the abutment was roughened again as described above to remove any resin residue from the crown fabrication and to optimize the bonding surface for better retention. In addition, this should create the same starting situation for all test specimens. Afterwards, the abutments were cleaned with 98% ethanol and dried completely with oil-free air. The bonding of the test specimens took place under the same environmental conditions at a room temperature of 24 °C by two dentists from the Clinic for Prosthetic Dentistry. The materials were stored in accordance with the manufacturer’s instructions. If the materials were stored in the refrigerator, they were brought to room temperature about 20 min before bonding and then prepared as instructed by the manufacturer and mixed in the specified ratio. Luting materials were applied in the marginal area of the crown lumen before the crowns were placed on the titanium abutments with finger pressure. Excess material was immediately and carefully removed with a dental probe to ensure a constant flow of excess material. A force of 50 N was applied to the test specimens throughout the curing period. For this purpose, the test specimens were placed in a special device after bonding. This device contains a weight of 5 kg, which loads the bonded test specimens from the occlusal surface of the temporary crown. To guarantee complete curing, the time for seating as defined by the manufacturer was doubled, considering that these times are usually specified for mouth temperature. For all methacrylate-based materials for which light-curing was indicated as being optional by the manufacturer, light-curing was carried out.

In order to investigate the influence of initial, short-term and long-term intraoral use of provisional restorations on the bond strength of titanium abutments, each test series (one for each provisional luting material) was divided into three subgroups (n = 10) and stored for the three different periods of provisional treatment time in distilled water. The individual subgroups were stored in distilled water for either 24 h (initial bond strength, subgroup 1), seven days (bond strength after short-term storage/use, subgroup 2) or 12 h (bond strength after long-term storage/use), with the latter followed by an additional thermocycling with 5000 cycles (subgroup 3). For thermocycling, the samples were alternately immersed for 20 s in 5 °C cold or 55 °C hot water with a dwell time of 5 s in between.

**Figure 5:**
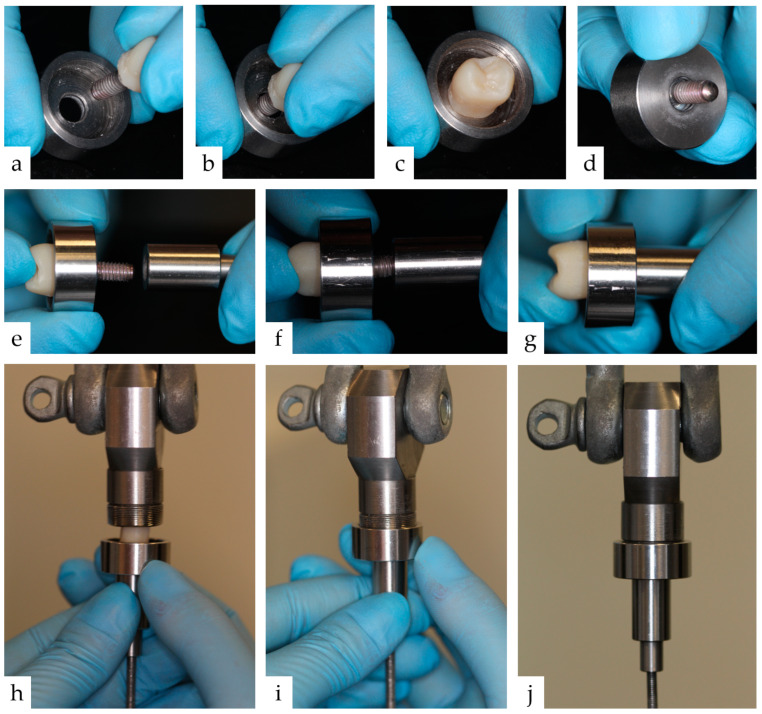
This figure shows the detailed procedure for fixing the test specimen in the test machine. (**a**) The metal mounting box has a central opening slightly larger than the implant diameter. This is slipped over the external thread of the implant (**b**) until there is contact with the crown (**c**). Slippage of the entire test specimen is prevented (**d**) by overcontouring the crown by approximately 2 mm in the marginal area. The external thread of the implant is used to fix the test specimen to the lower part of the testing machine. The implant is screwed into the testing machine (**e**–**g**). The internal thread of the mounting box, also screwed on (**h**–**j**), is then used to fix the test specimen to the upper part of the testing machine. When the test specimen is completely attached, the crown is no longer visible.

For bond strength testing, a specially proprietary-developed experimental setup was selected. It has been described in detail by Blender et al. [20]. As with the clinical procedure of provisional crown removal, shear and tensile forces occur simultaneously during this procedure. In addition, no further materials are required to fix the test specimen into the universal testing machine (Z010, Zwick/Roell, Ulm, Germany): for example, a material for bonding the implant to the testing machine. For the fixation of the test specimen to the lower part of the testing machine, the external thread of the implants is used and the upper fixation is created by a metal holding box with a diameter minimally larger than the implant diameter, but with a smaller diameter than the marginal area of the crown, so that the crown part of the assembled crown-implant specimen will not slip out of the metal box. A detailed description of the fixing of the test specimen is shown in Figure 5. The detachment of the single-tooth crowns took place at a rate of 0.5 mm/min until the maximum force in N for removal was reached. The value was documented in the test software (testeXpert III, Version 1.61, Zwick/Roell, Ulm, Germany). A fracture test was performed at a defined force threshold of 20 N. The maximum force achieved during debonding was then used to calculate the force per bonding surface between the crown and abutment, resulting in the bond strength in MPa. Therefore, the maximum force in N was divided by the bond area of 65.93 mm^2^ to obtain the bond strength in MPa. The bonding area was 65.93 mm^2^ based on the construction dimensions as specified by the manufacturer.

The data obtained were statistically analyzed descriptively (mean, standard deviation, 95% confidence interval (CI), median, minimum and maximum) using appropriate software (IBM SPSS Statistics 28, Armonk, NY, USA). Data were analyzed for their normal distribution using the Shapiro–Wilk test. To evaluate significant differences in bond strength between the materials or material-specific differences after the different storage scenarios, a single-factor analysis of variance with a subsequent post hoc test (Tukey or Games–Howell) was performed. A qualitative analysis of the fracture behavior of the luting materials was then carried out using a light microscope (Leica MS 5, Leica Camera AG, Wetzlar, Germany).

## 3. Results

For statistical evaluation of the data and visual fracture pattern analysis, all 270 specimens could be used. The mean and standard deviation of the bond strength (MPa) and retention force (N) of all nine temporary materials according to the different storage scenarios are described in Table 2.

For the further statistical analyses, the individual test subgroups (n = 10) were examined for normal distribution using the Shapiro–Wilk test. Only the subgroup of TBeu after 7 days did not show a normal distribution (*p* = 0.008). All other subgroups showed a normal distribution (*p* > 0.05).

### 3.1. Simulated Initial Bond Strength after 24 h of Storage in Distilled Water

**Figure 6:**
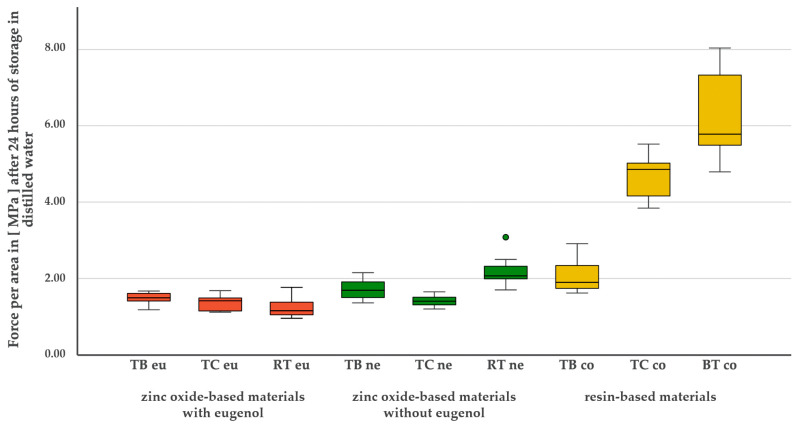
Bond strength values [MPa] of the nine tested temporary luting materials after 24 h of storage in distilled water.

A normal distribution was found for all materials after 24 h (*p* > 0.05). The test for variance homogeneity was highly significant (*p* < 0.001). After 24 h of storage in distilled water, two of the three resin-based materials (BTco: 6.19 MPa ± 1.08; TCco: 4.68 MPa ± 0.54) showed significantly higher bond strength values compared to the other materials (*p* < 0.001). The difference between the two materials was also significant (*p* = 0.031). For the third resin-based material (TBco: 2.03 MPa ± 0.40), the bond strength values were comparable to those of two representatives of the eugenol-free zinc oxide cements (RTne: 2.18 MPa ± 0.38; TBne: 1.71 MPa ± 0.26). RTne (2.18 Mpa ± 0.38) even showed a higher bond than TBco (2.03 MPa ± 0.40), but this was not significant (*p* = 0.993). TBco and RTne showed significantly better bond strength values compared to all eugenol-containing materials: TBeu (*p* = 0.030; *p* = 0.004), TCeu (*p* = 0.008; *p* < 0.001), RTeu (*p* = 0.003; *p* < 0.001), as well as TCne (*p* = 0.014; *p* = 0.002). TBne only showed a significantly better bond compared to RTeu (*p* = 0.028). No intragroup differences were observed in the group of eugenol-containing materials. A graphical representation of the bond strengths of the materials is shown in Figure 6.

### 3.2. Simulated Short-Term Bond Strength after Seven Days of Storage in Distilled Water

**Figure 7:**
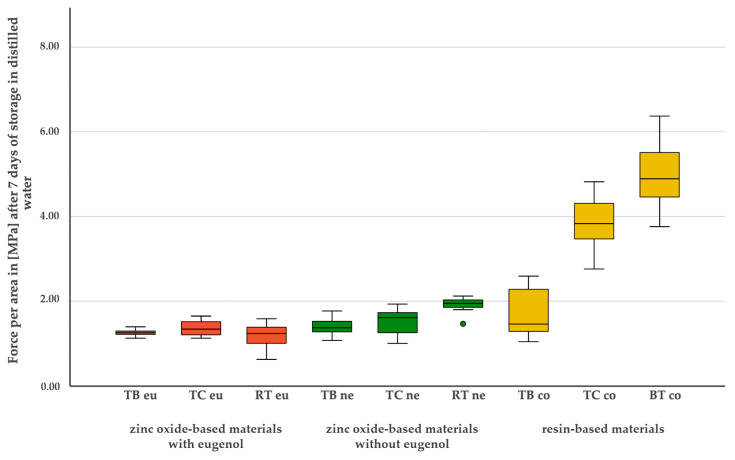
Bond strength values [MPa] of the nine tested temporary luting materials after seven days of storage in distilled water.

A normal distribution was found for eight of the nine materials after 7 days (*p* > 0.05). TBeu did not show a normal distribution (*p* = 0.008). The test for variance homogeneity was highly significant (*p* < 0.001). After seven days of storage in distilled water, an intergroup arrangement was observed: BTco and TCco continued to show highly significant better adhesion values compared to all other materials (*p* < 0.001), with the difference between the two materials still being significant (*p* = 0.033). The third resin-based material TBco showed a similar bond strength to all zinc oxide-based materials. While no significant differences were observed in the group of eugenol-containing zinc oxide cements, a significant difference was observed in the eugenol-free zinc oxide cement group: RTne compared to TBne as well as to all eugenol-containing materials (*p* < 0.001). Similar to the 24 h storage in distilled water, no differences were observed between the eugenol-containing materials. Figure 7 shows the descriptive values of the bond strength of all nine materials after seven days of storage in distilled water.

### 3.3. Simulated Long-Term Bond Strength after 12 h of Storage in Distilled Water Followed by Thermocycling

**Figure 8:**
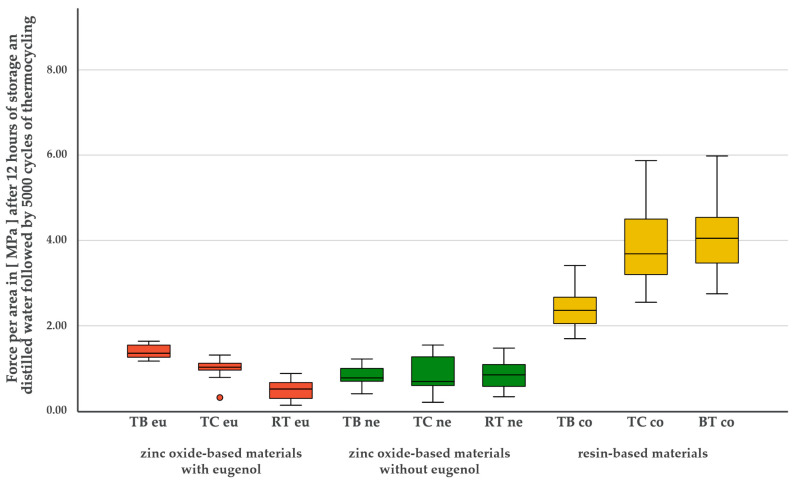
Bond strength values [MPa] of the nine tested temporary luting materials after 12 h of storage in distilled water and after artificial aging by thermocycling (5000 cycles).

A normal distribution was found for all materials after 12 h of storage in distilled water followed by artificial aging (*p* > 0.05). The test for variance homogeneity was highly significant (*p* < 0.001). After 12 h of storage in distilled water followed by artificial aging, all three resin-based materials showed the highest bond strength compared to all other materials (*p* < 0.001). While no difference was seen between BTco and TCco, both materials continued to have a significantly better bond strength than TBco (TCco: *p* = 0.031; BTco: *p* = 0.007). The materials of the eugenol-free zinc oxide cements showed no intragroup differences. However, significant differences were observed between all eugenol-containing materials (TBeu-Tceu: *p* = 0.030; TBeu-RTeu: *p* < 0.001; TCeu-RTeu: *p* = 0.011). TBeu not only showed a significantly higher bond strength compared to TCeu and RTeu, but also to TBne (*p* < 0.001), TCne (*p* = 0.050) and RTne (*p* = 0.026). Figure 8 shows the bond strengths of the materials after artificial aging.

### 3.4. Performance of the Individual Luting Materials

Six of the nine materials showed the lowest bond strength after 12 h of storage and artificial aging (BTco, RTeu, TCeu, TBeu, RTeu, TCeu, TBeu); the other three materials showed the lowest strength after 7 days of water storage (TCco, TBco, TBeu). For six materials, the highest bond values were observed after 24 h of storage (BTco, TCco, Rteu, TBeu, RTne, TBne); for two materials, the highest values were observed after seven days (TCeu; TCne). TBco was the only material to show the highest bond after 12 h of water storage and artificial aging. The artificial aging was able to significantly improve the bond strength of TCco and TBco compared to the individual storage scenarios, and was significantly higher for TBco (*p* = 0.009). Significant differences between the individual materials for the individual storage conditions can be seen in Table 3.

**Figure 9:**
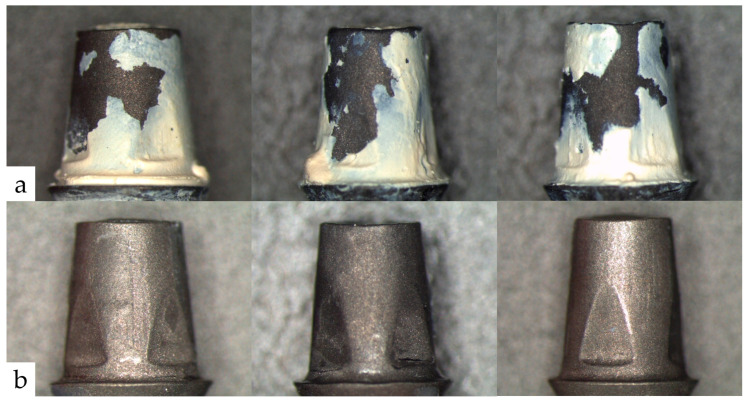
This figure gives examples of the abutment surface after removal of the crown. Figure (**a**) illustrates abutment surfaces with residual luting material. The remaining material is located at the inner surface of the crown. This mixed adhesive fracture pattern was observed for all zinc oxide cements, regardless of the type of storage. In contrast, Figure (**b**) shows abutment surfaces without any residual luting material. In this case, the entire material is located on the inner surface of the crown. This fracture type was consistently observed for all resin-based materials.

For all materials, an exclusively adhesive fracture pattern between them and the two surfaces—crown inside and abutment surface—could be observed. Zinc oxide-based materials showed a mixed adhesive fracture pattern, independent of their liquid component. In the case of composite-based materials, the entire luting material was found in the crown lumen; in some cases, the occlusal composite seal of the abutment was also removed during decementation (Figure 9).

## 4. Discussion

Differences in bond strength between the luting materials investigated in this study were observed for all three storage scenarios. Materials with a comparable or similar composition also tended to show comparable bond strength. Nevertheless, some significant differences were observed between the individual representatives within a material class. Other studies also reported heterogeneous results when looking at the bond strength of different temporary luting materials, depending on their ingredients [21,22,23,24,25,26,27,28]. A direct comparison with the results from other studies can only be made approximately, as the combination of temporary bis-acrylate single-tooth crowns, prefabricated titanium implant abutments, luting materials and the abutment surface selected here was not examined this way in any other study.

In our study, the resin-based materials achieved higher bond strengths after all three storage scenarios compared to the zinc oxide-based materials. The difference between BifixTemp and TempoCem ID (resin-based materials) and all other materials showed to be significant in all test series (*p* < 0.001), while the difference between TempBondClear (a resin-based material) and the eugenol-containing and the eugenol-free zinc oxide cements was significant only after the simulation of the long-term provisional restoration period.

In studies with a similar study design, in which both zinc oxide-based and resin-based temporary luting materials were examined, a dominance of the bond strength of the group of resin-based materials was observed, regardless of the storage conditions of the test specimens [21,22,26]. This increased bond strength compared to zinc oxide-based materials can be explained by the different compositions of the materials. Zinc oxide materials exhibit high water solubility, which is associated with lower compressive and tensile properties against pull-off forces [7]. In addition, the classic zinc oxide cements achieve their bond strength by mechanical components only, whereas the resin-based materials can additionally interact with other surfaces directly by adhesion [15]. This fact is confirmed by the results of the fracture analysis, which demonstrated a complete adhesive fracture behavior between the titanium surface and the luting material. For the composite-based materials, the luting material was found exclusively in the crown lumen in all cases, which can be attributed to direct interaction with the temporary bis-acrylate single-tooth crowns. The results for retention of the zinc oxide-based materials were comparable to the results for bond strength in other studies [29,30,31,32]. However, a trend to higher bond strengths for eugenol-free materials after simulation of an initial or short-term temporary restoration period can only be observed for certain materials in this group, which was also observed in studies with a similar study design [33].

Intragroup differences in bond strength between the individual representatives within a material class were observed, especially for the resin-based materials. These differences were almost consistently significant, which suggests that the different ingredients of the various manufacturers have a major impact on the adhesion behavior. In consequence, individual properties of certain luting materials could be modified by a specific composition of their ingredients. Similar studies on various resin-based materials support this assumption [22,25,26,27]. Within the group of zinc oxide-based materials, this causality was observed less frequently. Only in some cases were significant differences found between the materials within the group of eugenol-free zinc oxide materials, mainly in connection with increased bond strength values for RelyXTemp NE. In this group of materials, the composition of the individual ingredients appears to be less variable independently of the manufacturer.

There are no defined guideline values for a sufficient temporary bond strength. The temporary luting materials should be strong enough to resist functional forces for a certain period of time, but also weak enough to allow easy removal [34]. Values for the removal force between 40 and 200 N can be found in the literature, which have been confirmed by similar studies with temporary luting materials [22,35]. Due to the lack of standardization of these in vitro test procedures, even slight changes in the test setup can lead to major changes in the results [36,37]. To categorize the results obtained in this study, additional investigations examining the bond strength of permanent luting materials can be included [20,22,38]. Zinc oxide-based materials demonstrated comparable bonding behavior within their material class, whereas the bond strengths of the resin-based materials examined were very high after simulation of the initial and short-term temporary restoration period compared to the other material classes.

The influence of the three different simulated provisional restoration periods showed a significant influence on the individual performance of all materials. The higher water solubility of zinc oxide-based materials compared to resin-based materials may explain the difference in performance after the different storage times. The washout effect of the zinc oxide-based materials could be observed especially after artificial aging, as the bond strength values were significantly reduced compared to the results after 24 h or seven days of storage in distilled water. Also, the comparison of the values between 24 h and seven days of storage showed a reduction in bond strength. One possible explanation could be that the storage time of 7 days is probably still too short to significantly foster the solubility processes. The initial water absorption leads to an expansion of the luting material, which could be associated with increased filling of the cement gap and improved mechanical retention [38]. Resin-based materials showed a quite different and less predictable behavior depending on the respective luting material. The bond strength can not only be reduced but also increased by thermocycling [39,40,41]. Dähne et al. determined a similar behavior of the bond strength values after thermocycling with a resin-based and a eugenol-free provisional luting material [22]. While in the mentioned study after 24 h the bond strength of the composite-based material was significantly higher (100.5 N ± 39.14 N) than that of the eugenol-free material (61.16 N ± 20.19 N), this difference was no longer significant after the reduction in the bond strength by thermocycling (21.69 N ± 13.61 N and 16.97 N ± 12.36 N) [22]. The results of our investigation of the mentioned storage periods can be found in Figure 5 and Figure 7. For the simulation of the long-term temporary restoration period, the test specimens were initially stored in distilled water for 12 h before thermocycling with 5000 cycles was performed. This number of cycles corresponds approximately to six months of intraoral use [42]. A limitation of this study is the lack of simulated masticatory loading: for example, by combining thermocycling with alternating dynamic loading [43,44,45,46,47].

To investigate the bond strength of temporary luting materials, an in vitro test setup for crown removal tests was selected according to the standard procedure [48]. In test setups used by other authors, additional fixation materials are commonly used to fix the test specimens in the universal testing machine. However, this means that not only the relevant bonding surfaces on the inside of the crown and the outside of the abutment are examined, but also those used to attach the test specimen in the testing machine, which compromises the integrity of the test setup. The direct use of the external thread of the implant and the application of the pull-off force to the marginal crown component of our test setup simulates a direct and purely axial direction of force similar to the clinical condition. The need for additional fixation materials can thus be avoided [47].

Fracture analysis did not reveal any fractures within the implant or the provisional restorative material. This could be caused by the overcontouring of the restoration in the marginal area in favor of the purely axial pull-off direction in the test setup. In general, purely adhesive fractures were observed between the luting materials and the bonding surface of the abutments, which correlates with the relatively low bond strength values described in the literature [49]. However, this is also to be preferred for temporary luting materials, as it allows for easy removal of the restoration and facilitates the cleaning of the restoration and the bonding surface from luting material residues [50]. Due to the limitations of the in vitro test setup, the transferability to the clinic must be critically evaluated [43,51,52,53,54].

Based on the results of the present study, it can be suggested that the high bond strength of the resin-based materials is suitable for long-term temporary restoration periods. At the same time, these materials exhibit comparatively high values for initial and short-term temporary bond strength. For these restoration periods, zinc oxide-based materials with their lower but still sufficient bond strength values may be a reasonable alternative. An indication-based selection of the temporary luting material, depending on the planned treatment duration and the according duration of the provisional phase, is recommended.

## 5. Conclusions

Considering the limitations of this experimental setup, the following can be stated:

(1) Under in vitro conditions, two of the three resin-based materials can produce a stronger bond between temporary single-tooth crowns made of bis-acrylate and titanium abutment surfaces than conventional zinc oxide-based cements, regardless of the storage time or other artificial aging. However, there are major differences to be found between the individual materials of the resin-based material class, which can be attributed to their different ingredients and composition. Zinc oxide-based materials demonstrate a more consistent performance in this respect;
(2) Zinc oxide cements show a significant loss of retention during the simulated long-term provisional restoration period compared to initial bond strength and simulated short-term intraoral use. This could not be observed with the resin-based materials. Here, very inhomogeneous results were observed between the three materials: while one material showed the lowest bond strength after long-term temporary storage, one material showed the highest bond strength. The third material, on the other hand, showed a better bond strength compared to short-term temporary storage, but a lower bond strength compared to initial storage.

## Figures and Tables

**Table 1:** This table provides an overview of the three material classes to be investigated and the products selected for this purpose with their manufacturer and their delivery form. In addition, the abbreviation of the materials used in this study is indicated.

Material Class Based on the Components	Name, Manufacturer	Abbreviation	Dosage Form
zinc oxide-based temporary luting materials with eugenol	Temp Bond, Kerr Corporation, Orange, CA, USA,	TBeu	2 tubes, manual mixing
	TempoCem, DMG GmbH, Hamburg, Germany	TCeu	Smartmix syringe
	3M RelyX Temp E, 3M ESPE, Neuss, Germany	RTeu	2 tubes, manual mixing
zinc oxide-based temporary luting materials without eugenol	Temp Bond NE, Kerr Corporation, Orange, CA, USA	TBne	2 tubes, manual mixing
	TempoCemNE, DMG GmbH, Hamburg, Germany	TCne	Smartmix syringe
	3M RelyX Temp NE,3M ESPE, Neuss, Germany	RTne	2 tubes, manual mixing
resin-based temporary luting materials	Temp Bond Clear, Kerr Corporation, Orange, CA, USA	TBco	Smartmix syringe
	TempoCem ID, DMG GmbH, Hamburg, Germany	TCco	Smartmix syringe
	Bifix Temp, VOCO GmbH, Cuxhaven, Germany	BTco	Smartmix syringe

**Table 2:** Mean value and standard deviation of the bond strength values (MPa) and the maximum retention forces (N) at the time of detachment of all nine temporary materials according to the different storage scenarios (initial, short-term, long-term storage/use).

		Mean (±Standard Deviation)		
		24 h	7 days	TC
TB eu	in MPa	1.48 (±0.16)	1.26 (±0.08)	1.39 (±0.15)
	in N	97.62 (±10.39)	83.00 (±5.14)	91.54 (±10.01)
TC eu	in MPa	1.37 (±0.19)	1.37 (±0.18)	1.00 (±0.28)
	in N	90.42 (±12.33)	90.37 (±11.97)	65.81 (±18.21)
RT eu	in MPa	1.26 (±0.27)	1.20 (±0.28)	0.50 (±0.25)
	in N	82.93 (±18.03)	78.73 (±18.72)	32.79 (±16.16)
TB ne	in MPa	1.71 (±0.26)	1.41 (±0.20)	0.81 (±0.23)
	in N	112.46 (±16.99)	92.67 (±13.01)	53.58 (±15.33)
TC ne	in MPa	1.42 (±0.14)	1.52 (±0.31)	0.84 (±0.44)
	in N	93.70 (±9.21)	100.47 (±20.15)	55.11 (±29.19)
RT ne	in MPa	2.18 (±0.38)	1.92 (±0.19)	0.86 (±0.37)
	in N	143.90 (±25.31)	126.51 (±12.55)	56.77 (±24.73)
TB co	in MPa	2.03 (±0.40)	1.71 (±0.57)	2.41 (±0.48)
	in N	134.00 (±26.30)	112.85 (±37.73)	159.40 (±31.63)
TC co	in MPa	4.68 (±0.54)	3.79 (±0.64)	3.92 (±1.09)
	in N	308.50 (±35.74)	249.90 (±42.09)	258.30 (±71.56)
BT co	in MPa	6.19 (±1.08)	4.94 (±0.74)	4.19 (±1.04)
	in N	407.80 (±71.10)	325.60 (±48.73)	276.10 (±68.66)

**Table 3:** Intraindividual differences in the nine materials investigated at the different times of investigation by using a one-factorial analysis of variance. The Tukey test was carried out for multiple comparisons.

	Intraindividual Differences between Different Storage Scenarios					
	24 h vs. 7 d		7 d vs. TC		24 h vs. TC	
	24 h in MPa	7 d in MPa	7d in MPa	TC h in MPa	24 h in MPa	TC in MPa
TBeu	1.48 (±0.16)	1.26 (±0.08)	1.26 (±0.08)	1.39 (±0.15)	1.48 (±0.16)	1.39 (±0.15)
	***p*** **=** **0.003** **		*p* = 0.099		*p* = 0.284	
TCeu	1.37 (±0.19)	1.37 (±0.18)	1.37 (±0.18)	1.00 (±0.28)	1.37 (±0.19)	1.00 (±0.28)
	*p* = 1.000		***p*** **=** **0.002** **		***p*** **=** **0.002** **	
RTeu	1.26 (±0.27)	1.20 (±0.28)	1.20 (±0.28)	0.50 (±0.25)	1.26 (±0.27)	0.50 (±0.25)
	*p* = 0.863		***p*** **<** **0.001** **		***p*** **<** **0.001** **	
TBne	1.71 (±0.26)	1.41 (±0.20)	1.41 (±0.20)	0.81 (±0.23)	1.71 (±0.26)	0.81 (±0.23)
	***p*** **=** **0.019** **		***p*** **<** **0.001** **		***p*** **<** **0.001** **	
TCne *	1.42 (±0.14)	1.52 (±0.31)	1.52 (±0.31)	0.84 (±0.44)	1.42 (±0.14)	0.84 (±0.44)
	*p* = 0.610		***p*** **=** **0.003** **		***p*** **=** **0.006** **	
RTne	2.18 (±0.38)	1.92 (±0.19)	1.92 (±0.19)	0.86 (±0.37)	2.18 (±0.38)	0.86 (±0.37)
	*p* = 0.187		***p*** **<** **0.001** **		***p*** **<** **0.001** **	
TBco	2.03 (±0.40)	1.71 (±0.57)	1.71 (±0.57)	2.41 (±0.48)	2.03 (±0.40)	2.41 (±0.48)
	*p* = 0.320		***p*** **=** **0.009** **		*p* = 0.202	
TCco	4.68 (±0.54)	3.79 (±0.64)	3.79 (±0.64)	3.92 (±1.09)	4.68 (±0.54)	3.92 (±1.09)
	***p*** **=** **0.046** **		*p* = 0.931		*p* = 0.097	
BTco	6.19 (±1.08)	4.94 (±0.74)	4.94 (±0.74)	4.19 (±1.04)	6.19 (±1.08)	4.19 (±1.04)
	***p*** **=** **0.020** **		*p* = 0.209		***p*** **<** **0.001** **	

* Due to a lack of homogeneity of variance (*p* = 0.02), the Games–Howell test was used for multiple comparison. For all other materials, the homogeneity of variance (*p* > 0.05) was given. ** *p* values highlighted in bold indicate a significant result in the comparison of the two groups.

## Data Availability

The original contributions presented in the study are included in the article, further inquiries can be directed to the corresponding author.

## Appendix A

**Table A1:** This table shows the temporary luting materials used, with detailed information about the ingredients listed by the manufacturer.

Material Class Based on the Components	Name, Manufacturer	Abbreviation Dosage Form
zinc oxide-based temporary luting materials with eugenol	Temp Bond, Kerr, GER	Base paste: zinc oxide, white mineral oil (petroleum) Catalyst paste: eugenol, zinc acetate dihydrate, resin, oligomers
	TempoCem, DMG, GER	Base paste: zinc oxide, paraffin, additives Catalyst paste: natural resins, eugenol, additives
	3M RelyX Temp E, ESPE, USA	Base paste: zinc oxide, white mineral oil (petroleum), petrolatum Catalyst paste: hydrogenated colophony, modified colophony, eugenol, silanized silica, aliphatic acid
zinc oxide-based temporary luting materials without eugenol	Temp Bond NE, Kerr, GER	Base paste: zinc oxide, white mineral oil Catalyst paste: octanoic acid, 2-ethoxybenzoic acid, (R)-p-mentha-1,8-diene, d-limonene
	TempoCemNE, DMG, GER	Base paste: zinc oxide, paraffin, additives Catalyst paste: natural resins, fatty acids, additives
	3M RelyX Temp NE, ESPE, USA	Base paste: zinc oxide, white mineral oil (petroleum), petrolatum Catalyst paste: modified colophony, aliphatic acid, silanized silica
resin-based temporary luting materials	Temp Bond Clear, Kerr, GER	Base paste: acrylate resin, 2-hydroxyethyl methacrylate, silica Catalyst paste: acrylate resin, α,α-dimethylbenzyl hydroperoxide, cumene hydroperoxide, 2,6-di-tert-butyl-p-cresol, silica
	TempoCem ID, DMG, GER	Dental glass, ethoxylated bisphenol A dimethacrylate, aliphatic urethane methacrylate, unsaturated polyester resin blend, triethylene-glycol-dimethacrylate, 2-hydroxyethyl methacrylate, silicium dioxide, additives
	Bifix Temp, VOCO, GER	Urethan-dimethacrylat, triethylenglycoldimethacrylat, catalyst
